# Draft genome dataset of *Streptomyces griseoincarnatus* strain R-35 isolated from tidal pool sediments

**DOI:** 10.1016/j.dib.2024.111235

**Published:** 2024-12-16

**Authors:** Danielle Dana Mitchell, Jo-Marie Vreulink, Alaric Prins, Marilize Le Roes-Hill

**Affiliations:** Applied Microbial and Health Biotechnology Institute, Cape Peninsula University of Technology, PO Box 1906, Bellville, Cape Town, 7530, South Africa

**Keywords:** Actinobacteria, Genomics, Marine, *Streptomyces*, Tidal pool

## Abstract

The marine isolate, *Streptomyces griseoincarnatus* strain R-35, was isolated from marine sediments collected from the Glencairn Tidal Pool, Table Mountain National Park, Cape Town, South Africa. The genomic DNA was sequenced using the Ion Torrent GeneStudio™ S5 platform, and the *de novo* assembly was performed using the SPAdes assembler on the Centre for High Performance Computing (CHPC) Lengau Cluster located at the CSIR, Rosebank, South Africa. The draft genome assembly consisted of 722 contigs totaling 7,625,174 base pairs and a G+C% content of 72.2 mol%. Genome completeness and genome contamination were determined as 99.12% and 0.92%, respectively. Genome annotations performed using the Rapid Annotation with Subsystem Technology (RAST) and the Bacterial and Viral Bioinformatics Resource Centre (BV-BRC) determined the presence of 7996 coding sequences (CDS), 63 transfer RNAs (tRNAs), and six ribosomal RNAs (rRNAs). A total of 2570 hypothetical proteins were assigned, and 5246 proteins were assigned to function. The phylogenomic positioning of *S. griseoincarnatus* strain R-35 was determined using the Type Strain Genome Server (TYGS) and was found to be related to *S. griseoincarnatus* JCM 4381^T^, with a digital DNA-DNA hybridisation (dDDH) value of 84.1%, and an OrthoANIu value of 98.22%. The CARD RGI algorithm on Proksee predicted the presence of 6,107 antimicrobial resistance (AMR) features, 27 biosynthetic gene clusters (BGCs) were predicted using antiSMASH, while 189 carbohydrate-active enzymes (CAZymes) were predicted using dbCAN3. The raw genome sequencing data has been submitted to the National Center for Biotechnology (NCBI) under the BioProject ID PRJNA1129156 (BioSample ID Accession Number: SAMN42145163; Short Read Archive (SRA) Accession: SRR29633055; https://www.ncbi.nlm.nih.gov/bioproject/PRJNA1129156).

Specifications Table

**Table:** 

Subject	Microbial Genomics
Specific subject area	Microbiology, Genomics, Biotechnology
Type of data	Raw and processed data (tables, graphs, figures)
Data collection	*Streptomyces griseoincarnatus* strain R-35 was isolated from marine sediments collected from the Glencairn tidal pool, Table Mountain National Park. Cultures were maintained on ISP2 prepared with artificial seawater. Genomic DNA was extracted from the strain using a ZymoResearch QuickDNA Fecal/Soil Microbe Prep Kit. The DNA library was prepped using the Ion 540™ Chef Kit and sequenced using an Ion GeneStudio™ S5 Prime system. A draft genome assembly was performed using SPAdes on the Centre for High Performance Computing Lengau Cluster, CSIR, Rosebank, South Africa. Downstream processing was performed using Quast, CheckM, RAST, BV-BRC, TYGS, ANI calculator, Proksee, and antiSMASH.
Data source location	The isolate, *Streptomyces griseoincarnatus* strain R-35 was isolated from marine sediment collected from the Glencairn Tidal Pool (co-ordinates -34.162481, 18.432022)*.* The isolate is maintained in the culture collection at the Applied Microbial and Health Biotechnology Institute (AMHBI), Cape Peninsula University of Technology, Cape Town, South Africa.
Data accessibility	Repository name: The raw genome sequence reads for Streptomyces griseoincarnatus strain R-35 is deposited and publicly available on NCBI under BioProject PRJNA1129156 with the BioSample accession SAMN42145163. Data identification number: The short read archive (SRA) is available on NCBI under accession number SRR29633055. Direct URL to data: https://www.ncbi.nlm.nih.gov/bioproject/PRJNA1129156 Secondary Data: Outputs from the bioinformatics tools applied are publicly available on the Mendeley Data Repository at the following link: 10.17632/4tykmn5gyp.3
Related research article	Not applicable

## 1 Value of the Data

• The dataset details a core genome analysis describing a marine actinobacterial isolate, *Streptomyces griseoincarnatus* strain R-35, isolated from a marine site not previously explored for actinobacteria.
• The dataset provides a potential genomic resource for exploring biosynthetic genes, including those encoding for antimicrobials and enzymes.
• The dataset is relevant to clinical and industrial biotechnology applications.
• The dataset expands our current taxonomic understanding of strains identified as *Streptomyces griseoincarnatus* and their geographical distribution.

## 2 Background

The genus *Streptomyces* is a large, phenotypically diverse bacterial genus in the phylum *Actinomycetota* and currently contains 730 validly published child taxa [1]. With some exceptions, typical morphological characteristics include extensive branching substrate and aerial mycelia [2]. These organisms typically have large, linear genomes ranging from 8.5 to 12Mbp in size and high G+C content of between 67 and 78% [3]. *Streptomyces* species are abundant in various soil environments where they play a key role in the biogeochemical cycle but have also been extensively described in marine environments as free-living organisms or as symbionts [4]. *Streptomyces* species are prolific producers of secondary metabolites, accounting for about 17% of all known active secondary metabolites [5]. These secondary metabolites exhibit various properties, including antimicrobial, anti-inflammatory, antiviral, antitumour, and anti-diabetic activities [6], largely attributed to many biosynthetic gene clusters encoded for in their large genomes [7]. As part of a larger coastal biodiversity survey along the Western Cape, South Africa, *Streptomyces griseoincarnatus* strain R-35 was isolated from marine sediments collected from the Glencairn tidal pool, Table Mountain National Park and explored for its biosynthetic potential.

## 3 Data Description

The raw genome sequence data is deposited on NCBI under BioProject at accession PRJNA1129156.

At the root of the secondary data (analyses outputs) folder, a summary document of file and folder structure can be found (*file: R-35_Analyses_DirectoryContents.txt*)

The draft genome assembly of *Streptomyces griseoincarnatus* strain R-35 (*file: 001_Assembly > R-35_gDNA_assembly.fasta*) contains 722 contigs, with the largest contig being 157,132bp in size. The draft genome is 7,625,174bp in length with a G+C content of 72.2 mol%, and an N_50_ score of 25 615bp (*file: 002_Quast > R-35_QUAST.html*). The completeness and contamination of the genome of strain R-35 were 99.12% and 0.92%, respectively (Table 1, *file: 003_CheckM > tab_text > CheckM_summary_table.tsv*).

**Table 1:** Summary of draft genome assembly metrics for *Streptomyces griseoincarnatus* strain R-35.

Genome length (bp)	7,625,174
Number of contigs	722
G+C mol%	72.2
N_50_ (bp)	25,615
Marker lineage (CheckM)	f__*Streptomycetaceae*
Completeness (%)	99.12
Contamination (%)	0.92

**Fig. 1:**
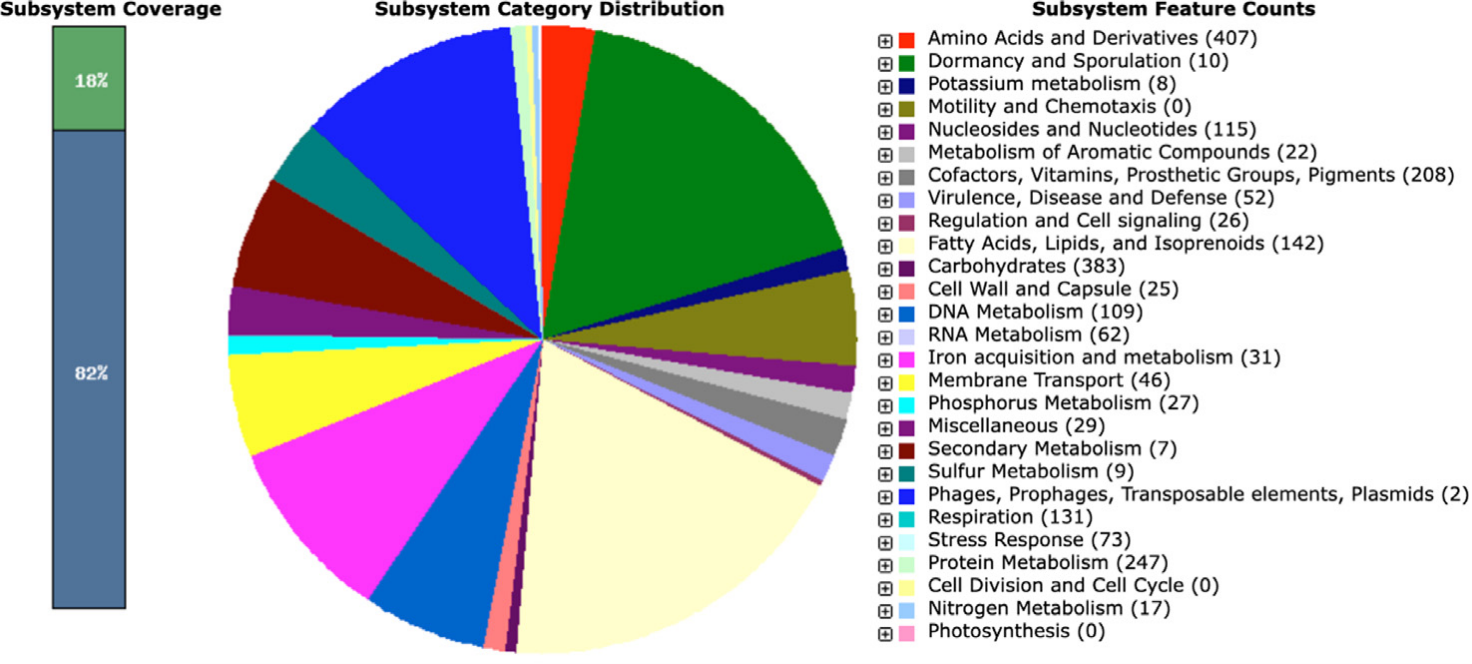
Subsystem of the draft genome of *Streptomyces griseoincarnatus* strain R-35 annotated through the RAST Annotation Server.

Genome annotations with the Rapid Annotation with Subsystem Technology (RAST) tool and the Bacterial and Viral Bioinformatics Resource Centre (BV-BRC) determined the presence of 7,996 coding sequences (CDS), 63 transfer RNA (tRNA) genes, and six ribosomal RNA (rRNA) genes (*full RAST feature list file: 004_Annotation > R-35_RAST.xls/gbk*). A total of 312 subsystem features were annotated, contributing 18% of the detected features (Fig. 1).

The BV-BRC annotation included 2570 hypothetical proteins and 5426 proteins assigned to function (Table 2). Of these, 1148 proteins were assigned with Enzyme Commission (EC) numbers, 1213 with Gene Ontology (GO) assignments, and 1113 with KEGG pathway assignments. The BV-BRC annotation also includes two protein families – genus-specific protein families (PLFams) and cross-genus protein families (PGFams), of which there were 7307 and 7402, respectively (*file: 004_Annotations > R-35_FullGenomeReport_PATRIC.html*).

**Table 2:** Protein features annotated in BV-BRC for draft genome assembly of *Streptomyces griseoincarnatus* strain R-35.

Feature	Total
Hypothetical Proteins	2,570
Proteins with functional assignments	5,426
Proteins with EC number assignments	1,418
Proteins with GO assignments	1,213
Proteins with KEGG pathways assignments	1,113
Proteins with PATRIC genus-specific family (PLFam) assignments	7,307
Proteins with PATRIC cross-genus family (PGFam) assignments	7,402

**Fig. 2:**
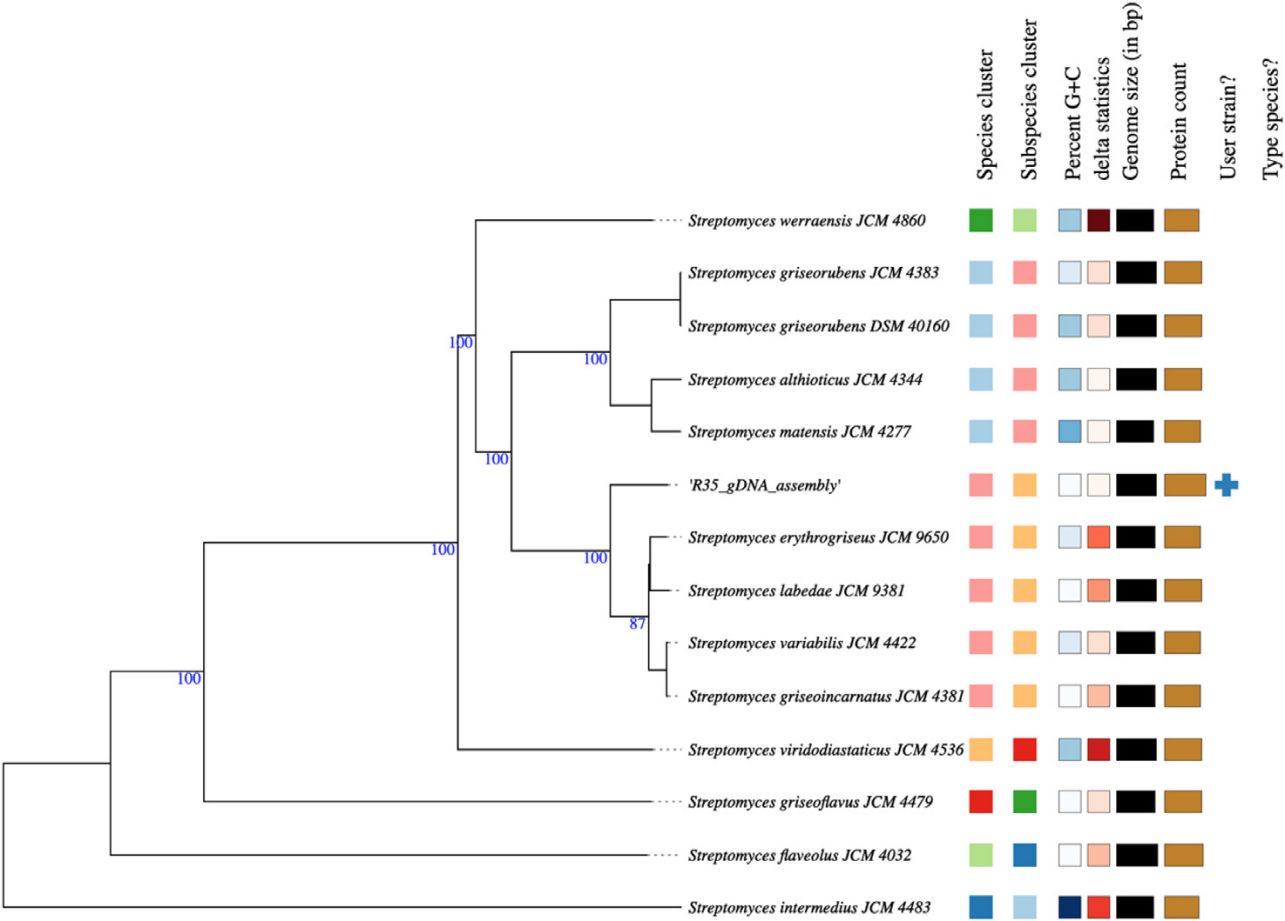
Phylogram illustrating the relatedness of *Streptomyces griseoincarnatus* strain R-35 to other known *Streptomyces* strains.

The phylogenomic positioning of *S. griseoincarnatus* strain R-35 was determined using the Type Strain Genome Server (TYGS) (Fig. 2). Strain R-35 is closely related to *Streptomyces erythrogriseus* JCM 9650^T^, *Streptomyces labedae* JCM 9381^T^, *Streptomyces griseoincarnatus* JCM 4381^T^, and *Streptomyces variablilis* JCM 4422^T^. A digital DNA-DNA hybridization (dDDH) value of 84.1% and a minimal G+C % difference of 0.3 assigned strain R-35 as *S. griseoincarnatus* (*file: 005_Phylogenomics > R-35_TYGS_JobResults.pdf*). As confirmed by the OrthoANIu value (98.2%) against *S. griseoincarnatus* JCM 4381^T^ (*file: 005_Phylogenomics > R-35_OrthoANIu_results.pdf)*.

Several specialty genes were annotated in BV-BRC with homology to known transporters, virulence factors, drug targets, and antibiotic resistance genes. Table 3 summarises homologous gene searches against several databases, including CARD, NDARO, DrugBank, and Victors.

**Table 3:** Summary of specialty genes present in the genome of *Streptomyces griseoincarnatus* strain R-35 as predicted using BV-BRC.

Type of Gene	Source Database	Amount
Antibiotic Resistance	CARD	6
Antibiotic Resistance	NDARO	1
Antibiotic Resistance	PATRIC	52
Drug Target	DrugBank	10
Drug Target	TTD	1
Transporter	TCDB	61
Virulence Factor	PATRIC_VF	8
Virulence Factor	Victors	2

**Fig. 3:**
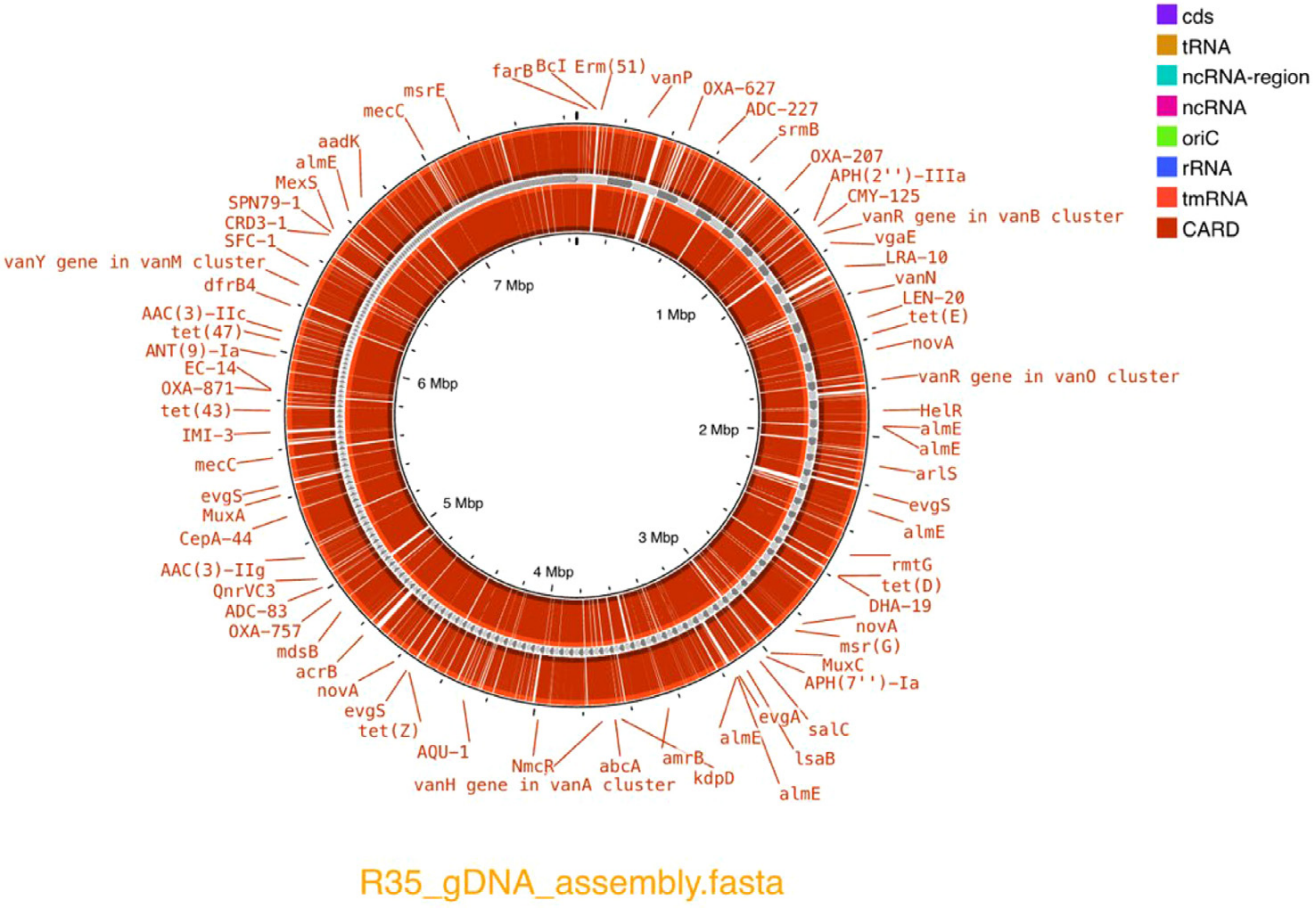
Filtered circular map of antimicrobial resistance (AMR) genes detected in the draft genome of *Streptomyces griseoincarnatus* strain R-35 constructed in Proksee.

Furthermore, antimicrobial resistance (AMR) genes were searched for using Proksee (CARD RGI) by including partial hits to compensate for shorter contigs in the genome assembly. A total of 6,107 AMR features were detected (*file: 004_Annotations > R-35_AMR_CARD_Proksee_cgview.json*). Several vancomycin and tetracycline resistance genes were observed (Fig. 3).

**Fig. 4:**
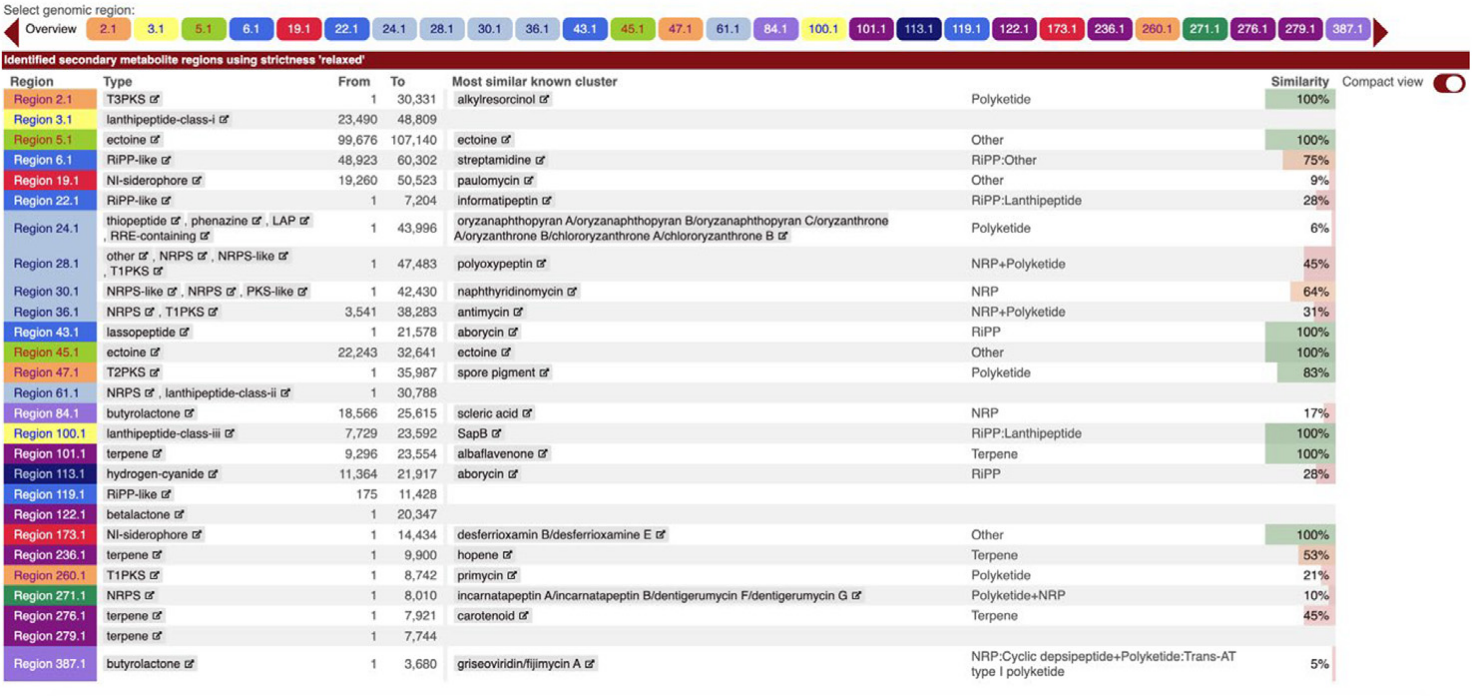
antiSMASH output of biosynthetic gene clusters detected in the draft genome assembly of *Streptomyces griseoincarnatus* strain R-35.

The presence of biosynthetic gene clusters (BGCs) was determined using antiSMASH (*file: 006_antiSMASH > index.*html). A total of 27 BGCs were detected, predominantly polyketide synthases (PKS – type 1, type 2, and type 3), non-ribosomal peptide synthetase (NRPSs), ribosomally synthesized and post-translationally modified peptide (RiPP)-like, terpenes and lactones (Fig. 4). Sequence homology ranged from 5 to 100% similarity to known BCGs.

**Fig. 5:**
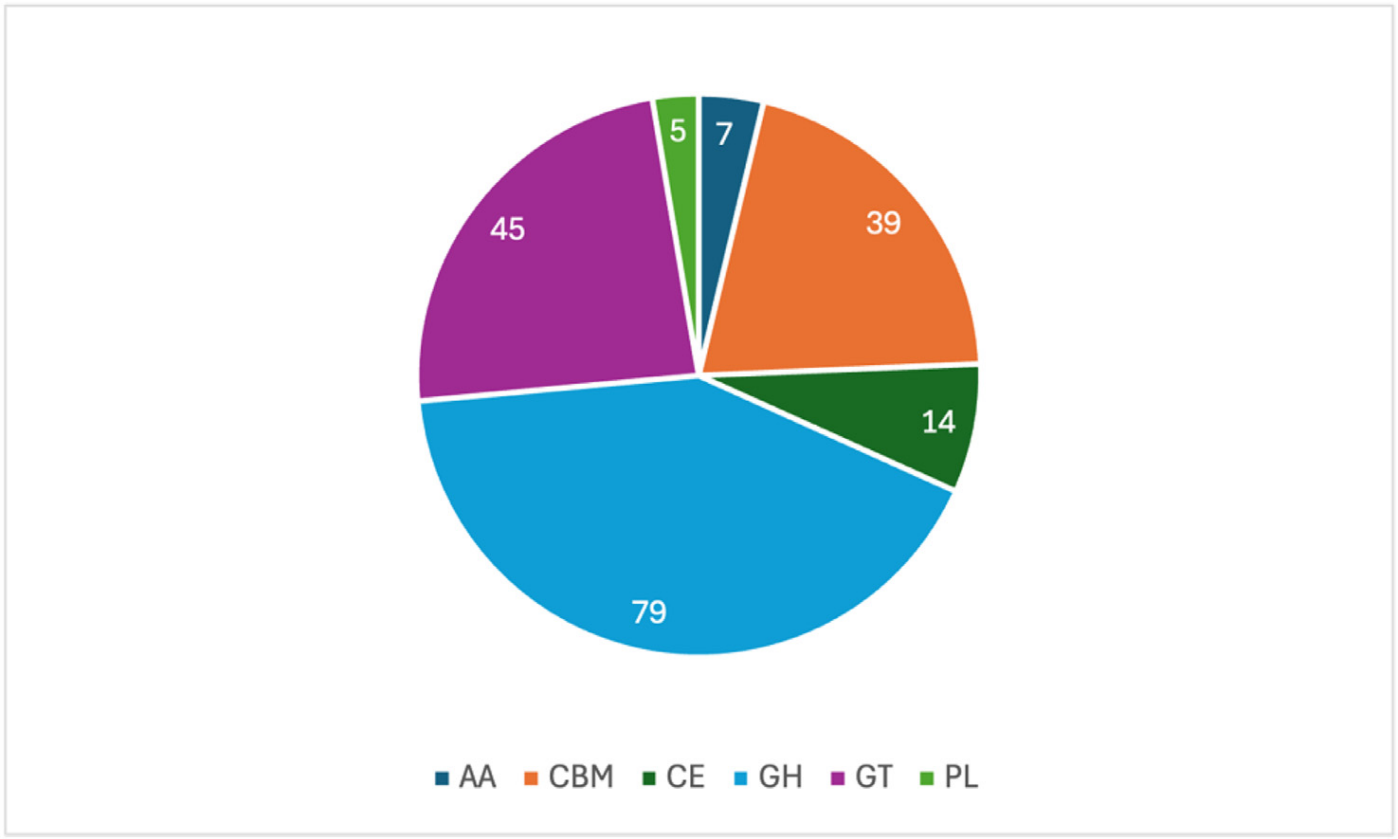
Distribution of carbohydrate-active enzymes (CAZymes) detected in the draft genome of *Streptomyces griseoincarnatus* strain R-35 using dbCAN3. Data is filtered to include CAZymes only confirmed by all three algorithms. AA = auxiliary activities; CBM = carbohydrate-binding modules; CE = carbohydrate esterases; GH = glycoside hydrolases; GT = glycosyltransferases; PL = polysaccharide lyases.

In addition to BGCs, carbohydrate-active enzymes (CAZymes) were determined using dbCAN3. Assignments were filtered by the predictions made by all three algorithms used. A total of 189 CAZymes were detected by all three algorithms (*file: 007_CAZymes > R-35_CAZymes_Summary.txt*). The detected CAZymes include auxiliary activities (AA), carbohydrate-binding modules (CBM), carbohydrate esterases (CE), glycoside hydrolases (GH), glycosyltransferases (GT), and polysaccharide lyases (PL) (Fig. 5).

## 4 Experimental Design, Materials and Methods

All reagents were purchased from Merck-Millipore, South Africa, unless otherwise indicated. Genomic DNA was isolated from a 5-day-old liquid culture of *Streptomyces griseoincarnatus* strain R-35 cultivated in International *Streptomyces* Project medium No.2 (ISP2; g/L: 4.0 glucose; 10.0 malt extract; 4.0 yeast extract) prepared with artificial seawater (g/L: 38.2 Red Sea Salt, Red Sea™) (orbital shaking at 160rpm at 30°C). Cell mass was collected by centrifugation at 10,000rpm for 2 min. After the removal of the supernatant fluid, the cell mass was used for DNA extraction using the *Quick*-DNA Fecal/Soil Microbe Miniprep Kit (Zymo Research), according to the manufacturer's instructions. DNA integrity was confirmed by agarose gel electrophoresis (1%, w/v) in 1× Tris-Acetate-EDTA buffer, pH8.3, 100V for 60 min. The gel (containing 10µg/mL ethidium bromide) was visualized using a Gel Doc XR+ (Bio-Rad) gel documentation system.

The genomic DNA was submitted to the DNA Sequencing Unit at the Central Analytical Facility (CAF, Stellenbosch University, South Africa) for sequencing. The library preparation was performed in-house at CAF using the Ion 540™ Chef Kit. Sequencing was performed on the Ion Torrent GeneStudio™ S5 system using 200bp chemistry with a 100× coverage, assuming a genome size of 8-10Mbp. Raw sequence reads (.bam format) obtained from CAF were uploaded to the Lengau cluster at the Centre for High Performance Computing (CSIR, Rosebank, South Africa). Draft genome assembly was performed using the St. Petersburg Genome Assembler (SPAdes, version 3.15.4, [8]) using an IonTorrent flag (*file: 001_Assembly > R-35_gDNA_assembly.log*). The resulting contigs (.fasta format) were used for downstream processing.

Default parameters were used for all bioinformatics tools unless otherwise stated. The quality of the draft genome was determined using Quast (version 5.2.0, [9]). Genome completeness and contamination were analysed using CheckM (v1.10.18) [10] on the KBase webserver (https://kbase.us/, [11]) with advanced parameters “Reference tree = full tree” and “Save All Plots = Save”. The Type (Strain) Genome Server (https://tygs.dsmz.de/, [12]) and ANI calculator (https://www.ezbiocloud.net/tools/ani/, [13]) were used to determine the taxonomic assignment of strain R-35. For the OrthoANI calculation, the genome sequence of the type strain, *S. griseoincarnatus* JCM 4381^T^, was used for comparison with the R-35 genome. Genome annotations were performed on the Rapid Annotation using Subsystem Technology (RAST) webserver (https://rast.nmpdr.org/, [14]) using RASTtk, disabling the replication option, and specifying the use of the bacterial code for annotation. Additional annotations were performed with the Comprehensive Genome Analysis Tool on the BV-BRC webserver (https://www.bv-brc.org/app/ComprehensiveGenomeAnalysis, [15]) using the default parameters (“Assembly strategy” set to “Auto”; “Annotation recipe” set to “Bacteria / Archaea”). Additional annotations for antimicrobial resistance genes were obtained using the CARD Resistance Gene Identifier tool (version 6.0.3) on the Proksee webserver [16], enabling the options related to “loose hits”, “nudged hits” and “low sequence quality” to include shorter contigs in the draft genome assembly. The presence of biosynthetic gene clusters was determined using the antiSMASH webserver (version 7.1.0; https://antismash.secondarymetabolites.org/, [17]) on “relaxed” mode and enabling all additional features. Carbohydrate-active enzyme (CAZyme) annotations were obtained using the dbCAN3 webserver (sequence type was specified as “nucleotide sequence”, all tools, including “substrate prediction” were selected for the annotation) [18]. CAZymes were filtered based on the prediction by all three algorithms (HMMer, dbCAN_sub, and DIAMOND), as provided in the output table.

## Limitations

The use of short-read technology (Ion Torrent) for the *de novo* assembly of the draft genome resulted in the generation of many contigs (> 500). As such, some contigs may result in suboptimal downstream processing results, particular in the case of BGC annotation.

## Ethics Statement

The authors have read and followed the ethical requirements for publication in Data in Brief and hereby confirm that the current work does not involve human subjects, animal experiments, or any data collected from social media platforms.

## CRediT authorship contribution statement

**Danielle Dana Mitchell:** Investigation, Formal analysis, Conceptualization, Writing – review & editing. **Jo-Marie Vreulink:** Investigation, Formal analysis, Conceptualization, Writing – review & editing, Supervision. **Alaric Prins:** Investigation, Formal analysis, Conceptualization, Data curation, Writing – original draft. **Marilize Le Roes-Hill:** Investigation, Formal analysis, Conceptualization, Writing – review & editing, Supervision, Funding acquisition.

## Data Availability

Mendeley DataGenomics dataset of marine isolate Streptomyces griseoincarnatus strain R-35 (Original data). NCBIMulti-omics data from marine sites along the Western Cape, South Africa (Original data).

## References

[1] Parte A.C. (2014). LPSN—list of prokaryotic names with standing in nomenclature. Nucleic Acids Res..

[2] Chater K.F. (2016). Recent advances in understanding *Streptomyces*. F1000Res.

[3] Law J.W.-F., Tan K.-X., Wong S.H., Mutalib N.-S.A., Lee L.-H. (2018). Taxonomic and characterization methods of *Streptomyces*: a review. Prog. Micobes. Mol. Biol..

[4] Quinn G.A., Banat A.M., Abdelhameed A.M., Banat I.M. (2020). *Streptomyces* from traditional medicine: sources of new innovations in antibiotic discovery. J. Med. Microbiol..

[5] Chassagne F., Cabanac G., Hubert G., David B., Marti G. (2019). The landscape of natural product diversity and their pharmacological relevance from a focus on the dictionary of natural products®. Phytochem. Rev..

[6] Hassan S.S.U., Shaikh A.L. (2017). Marine actinobacteria as a drug treasure house. Biomed. Pharmacother..

[7] Alam K., Mazumder A., Sikdar S., Zhao Y.-M., Hao J., Song C., Wang Y., Sarkar R., Islam S., Zhang Y., Li A. (2022). *Streptomyces*: the biofactory of secondary metabolites. Front. Microbiol..

[8] Prjibelski A., Antipov D., Meleshko D., Lapidus A., Korobeynikov A. (2020). Using SPAdes De Novo assembler. Curr. Protoc. Bioinform..

[9] Gurevich A., Saveliev V., Vyahhi N., Tesler G. (2013). QUAST: quality assessment tool for genome assemblies. Bioinformatics.

[10] Parks D.H., Imelfort M., Skennerton C.T., Hugenholtz P., Tyson G.W. (2015). CheckM: assessing the quality of microbial genomes recovered from isolates, single cells, and metagenomes. Genome Res..

[11] Arkin A.P., Cottingham R.W., Henry C.S., Harris N.L., Stevens R.L., Maslov S., Dehal P., Ware D., Perez F., Canon S., Sneddon M.W., Henderson M.L., Riehl W.J., Murphy-Olson D., Chan S.Y., Kamimura R.T., Kumari S., Drake M.M., Brettin T.S., Glass E.M., Chivian D., Gunter D., Weston D.J., Allen B.H., Baumohl J., Best A.A., Bowen B., Brenner S.E., Bun C.C., Chandonia J.-M., Chia J.-M., Colasanti R., Conrad N., Davis J.J., Davison B.H., DeJongh M., Devoid S., Dietrich E., Dubchak I., Edirisinghe J.N., Fang G., Faria J.P., Frybarger P.M., Gerlach W., Gerstein M., Greiner A., Gurtowski J., Haun H.L., He F., Jain R., Joachimiak M.P., Keegan K.P., Kondo S., Kumar V., Land M.L., Meyer F., Mills M., Novichkov P.S., Oh T., Olsen G.J., Olson R., Parrello B., Pasternak S., Pearson E., Poon S.S., Price G.A., Ramakrishnan S., Ranjan P., Ronald P.C., Schatz M.C., Seaver S.M.D., Shukla M., Sutormin R.A., Syed M.H., Thomason J., Tintle N.L., Wang D., Xia F., Yoo H., Yoo S., Yu D. (2018). KBase: the United States department of energy systems biology knowledgebase. Nat. Biotechnol..

[12] Meier-Kolthoff J.P., Göker M. (2019). TYGS is an automated high-throughput platform for state-of-the-art genome-based taxonomy. Nat. Commun..

[13] Yoon S.H., Ha S.M., Lim J.M., Kwon S.J., Chun J. (2017). A large-scale evaluation of algorithms to calculate average nucleotide identity. Antonie van Leeuwenhoek.

[14] Aziz R.K., Bartels D., Best A.A., DeJongh M., Disz T., Edwards R.A., Formsma K., Gerdes S., Glass E.M., Kubal M., Meyer F., Olsen G.J., Olson R., Osterman A.L., Overbeek R.A., McNeil L.K., Paarmann D., Paczian T., Parrello B., Pusch G.D., Reich C., Stevens R., Vassieva O., Vonstein V., Wilke A., Zagnitko O. (2008). The RAST server: rapid annotations using subsystems technology. BMC Genom..

[15] Olson R.D., Assaf R., Brettin T., Conrad N., Cucinell C., Davis J.J., Dempsey D.M., Dickerman A., Dietrich E.M., Kenyon R.W., Kuscuoglu M., Lefkowitz E.J., Lu J., Machi D., Macken C., Mao C., Niewiadomska A., Nguyen M., Olsen G.J., Overbeek J.C., Parrello B., Parrello V., Porter J.S., Pusch G.D., Shukla M., Singh I., Stewart L., Tan G., Thomas C., VanOeffelen M., Vonstein V., Wallace Z.S., Warren A.S., Wattam A.R., Xia F., Yoo H., Zhang Y., Zmasek C.M., Scheuermann R.H., Stevens R.L. (2023). Introducing the bacterial and viral bioinformatics resource centre (BV-BRC): a resource combining PATRIC, IRD and ViPR. Nucl. Acids Res..

[16] Grant J.R., Enns E., Marinier E., Mandal A., Herman E.K., Chen C., Graham M., Van Domselaar G., Stothard P. (2023). Proksee: in-depth characterization and visualization of bacterial genomes. Nucl. Acids Res..

[17] Blin K., Shaw S., Augustijn H.E., Reitz Z.L., Biermann F., Alanjary M., Fetter A., Terlouw B.R., Metcalf W.W., Helfrich E.J.N., van Wezel G.P., Medema M.H., Weber T. (2023). antiSMASH 7.0: new and improved predictions for detection, regulation, chemical structures and visualisation. Nucl. Acids Res..

[18] Zheng J., Ge Q., Yan Y., Zhang X., Huang L., Yin Y. (2023). dbCAN3: automated carbohydrate-active enzyme and substrate annotation. Nucl. Acids Res..

